# Utility of shape evolution and displacement in the classification of chronic multiple sclerosis lesions

**DOI:** 10.1038/s41598-020-76420-8

**Published:** 2020-11-11

**Authors:** Darin T. Okuda, Tatum M. Moog, Morgan McCreary, Jennifer N. Bachand, Andrew Wilson, Katy Wright, Mandy D. Winkler, Osniel Gonzalez Ramos, Aiden P. Blinn, Yeqi Wang, Thomas Stanley, Marco C. Pinho, Braeden D. Newton, Xiaohu Guo

**Affiliations:** 1 Department of Neurology, Neuroinnovation Program, Multiple Sclerosis and Neuroimmunology Imaging Program, Clinical Center for Multiple Sclerosis, UT Southwestern Medical Center, 5323 Harry Hines Blvd., Dallas, TX 75390-8806 USA; 2 School of Medicine, UT Southwestern Medical Center, 6011 Harry Hines Blvd., Dallas, TX 75235 USA; 3 Department of Computer Science, University of Texas at Dallas, 800 W. Campbell Rd., Richardson, TX 75080 USA; 4 Washington University, 1 Brookings Dr., St. Louis, MO 63130 USA; 5 Department of Radiology, UT Southwestern Medical Center, 5323 Harry Hines Blvd., Dallas, TX 75019 U.S.A.; 6 Cumming School of Medicine, University of Calgary, 3330 Hospital Drive NW, Calgary, AB T2N 4N1 Canada

**Keywords:** Neuroscience, Neurology, Demyelinating diseases, Neurovascular disorders

## Abstract

The accurate recognition of multiple sclerosis (MS) lesions is challenged by the high sensitivity and imperfect specificity of MRI. To examine whether longitudinal changes in volume, surface area, 3-dimensional (3D) displacement (i.e. change in lesion position), and 3D deformation (i.e. change in lesion shape) could inform on the origin of supratentorial brain lesions, we prospectively enrolled 23 patients with MS and 11 patients with small vessel disease (SVD) and performed standardized 3-T 3D brain MRI studies. Bayesian linear mixed effects regression models were constructed to evaluate associations between changes in lesion morphology and disease state. A total of 248 MS and 157 SVD lesions were studied. Individual MS lesions demonstrated significant decreases in volume < 3.75mm^3^ (*p* = 0.04), greater shifts in 3D displacement by 23.4% with increasing duration between MRI time points (*p* = 0.007), and greater transitions to a more non-spherical shape (*p* < 0.0001). If 62.2% of lesions within a given MRI study had a calculated theoretical radius > 2.49 based on deviation from a perfect 3D sphere, a 92.7% in-sample and 91.2% out-of-sample accuracy was identified for the diagnosis of MS. Longitudinal 3D shape evolution and displacement characteristics may improve lesion classification, adding to MRI techniques aimed at improving lesion specificity.

## Introduction

The diagnosis of multiple sclerosis (MS) is based on both clinical and radiological features, requiring fulfillment of both dissemination in time and space criteria^1^. Central to the diagnosis are magnetic resonance imaging (MRI) features and T2-hyperintensities highly suggestive of inflammatory demyelination based on lesion size and location. However, white matter anomalies resulting from microvascular disease, migraine headache, and normal aging, a general category known as cerebral small vessel disease (SVD)^2^, may be misinterpreted as representing the “classic” MRI features of MS, resulting in misdiagnosis^3^. The introduction of novel imaging metrics enabled the visual appreciation of central venous vasculature within lesions or the identification of hypointense rims, improving the specificity of lesions related to MS^4–8^.

Within existing clinical trials in MS, the presence of new or enlarging T2 lesions is used as a metric to define disease advancement. In clinical practice, the identification of new MS lesions between MRI time points may have implications in the recommended treatment course, neuroimaging frequency, and clinical follow-up. Recently, the importance of slowly expanding or ‘smoldering’ lesions in chronic active disease has gained prominence, highlighting the importance of in vivo measures quantifying subtle changes in existing lesions^9–11^. More recently, the clinical importance of paramagnetic rims, supported by histopathological data demonstrating chronic active inflammation, in association with disability outcomes has been reported^12^. However, the identification of such changes in real world settings from conventional imaging studies remain below the level of detection for the human eye.

Exploring the value of new approaches focused on studying the evolution of structural characteristics of chronic MS lesions, specifically changes in 3-dimensional (3D) shape and size, along with shifts in lesion position over time may reveal unique transitions that improve upon our understanding of lesion behavior in comparison to white matter injury resulting from a non-autoimmune mechanism. In the present study, we characterize the evolution of 3D morphological and spatial characteristics of chronic MS lesions to those resulting from SVD between two MRI time points and determine if changes exist in volume, surface area, lesion displacement or shape that may further enhance our ability to classify disease.

## Results

The study cohort was comprised of 34 patients, 23 with MS (female: 14 (60.9%); mean age (standard deviation): 42.4 years (11.9 years), having a median disease duration of 1.99 years (25th–75th percentile: 0.54, 5.94 years)) and 11 with SVD (female: 11 (100%); 52.5 years (7.63 years)) yielding 405 lesions for study. Of the 11 SVD patients, chronic migraine headache history was present in 7 with the remaining 4 patients having an established diagnosis of hypertension. A total of 248 MS lesions and 157 SVD lesions were included in the analysis. Of the 23 MS patients studied, 18 were exposed to FDA approved disease modifying therapies (alemtuzumab: 1, dimethyl fumarate: 8, fingolimod: 2, glatiramer acetate: 1, natalizumab: 3, ocrelizumab: 2, teriflunomide: 1) with a median treatment exposure of 3.13 years (range: 0.30–15.39). Clinical relapses related to acute demyelinating events occurring within 90 days of MRI time points 1 and 2 were not observed in the MS group. The baseline demographic information, clinical characteristics of the groups studied, and lesion-level data by group are summarized within the Table 1.

**Table 1:** Summary of demographic, clinical, and lesion-level descriptive data from the study cohort.

	Multiple sclerosis	Small vessel disease
**Clinical data**		
Patients (n)	23	11
Age		
Mean (standard deviation)	42.4 years (11.9)	52.5 years (7.63)
Female (%)	14 (60.9)	11 (100)
Race (%)		
White	21 (91.3)	10 (90.9)
African American	2 (8.7)	0 (0)
Asian	0 (0)	2 (9.1)
Hispanic (%)	2 (8.7)	2 (18.2)
Disease duration		
Median (P_25_, P_75_)	1.99 years (0.54, 5.94)	–
Median Lesion Number (P_25_, P_75_)	11 (6.5, 14.5)	14 (12, 17)
**Lesion-level data**		
Lesions analyzed (n)	248	157
Duration between MRI studies		
Median (P_25_, P_75_)	1.65 years (1.26, 1.91)	2.74 years (1.72, 3.46)
Change in volume between MRI time points		
Median (P_25_, P_75_)	− 2.32 mm^3^ (− 9.67, 3.48)	3.94 mm^3^ (− 0.65, 10.1)
Change in surface area between MRI time points		
Median (P_25_, P_75_)	− 2.16 mm^2^ (− 8.19, 3.51)	4.18 mm^2^ (− 0.63, 8.93)
Theoretical Radius MRI Time Point 1 (R_*ij*1_)		
Median ((R_ij1_ − 1) $\times$ 100) (P_25_, P_75_)	3.38 (2.41, 4.63)	2.17 (1.54, 2.81)
Theoretical Radius MRI Time Point 2 (R_*ij*2_)		
Median ((R_ij2_ − 1) $\times$ 100) (P_25_, P_75_)	3.46 (2.48, 4.60)	2.10 (1.52, 3.06)
Displacement		
Median (P_25_, P_75_)	0.39 mm (0.28, 0.56)	0.32 mm (0.22, 0.42)

### Volume and surface area

At the individual lesion level, a reduction in lesion volume below 3.75 mm^3^ between MRI time points was observed in the MS cohort as compared to the SVD cohort (95% Credible Interval (CrI) = (− 7.60, − 0.12), Bayesian *p* value = 0.04). Although not statistically significant, we observed that the volume change between time points in the SVD cohort was largely positive (95% CrI = (− 0.34, 5.18), Bayesian *p* value = 0.08) while the volume change between time points in the MS cohort was largely negative (95% CrI = (− 3.55, 0.71), Bayesian *p* value = 0.18), controlling for age, volume of the given lesion at baseline, and duration between MRI studies. Regarding change in surface area between MRI time points, a significant difference was not observed between the two groups (95% CrI = (− 7.08, 0.46), Bayesian *p* value = 0.09). Positive surface area changes in the SVD group (95% CrI = (− 0.56, 5.18), Bayesian *p* value = 0.12) and negative changes in the MS cohort (95% CrI = (− 3.03, 1.13), *p* = 0.34) that did not reach statistical significance were identified after controlling for age, surface area of the given lesion at baseline, and duration between MRI studies.

### Displacement (change in lesion positioning)

**Figure 1:**
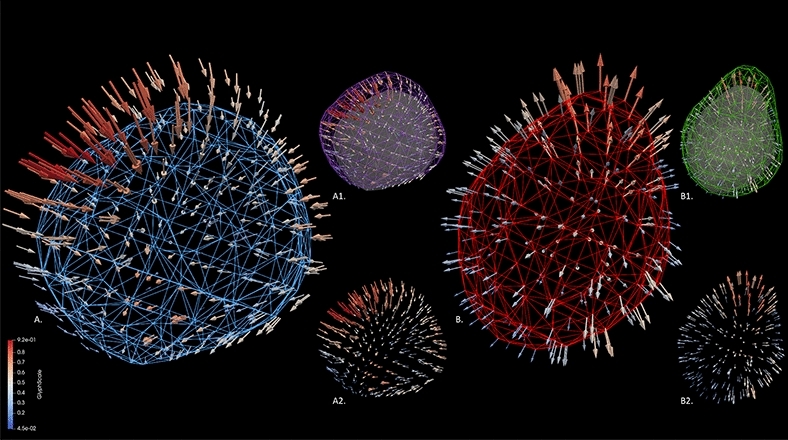
Three-dimensional (3D) displacement vectors from a (**A**) MS and (**B**) SVD lesion demonstrating both the magnitude (red indicating a higher degree of displacement) and direction (positioning of arrows) of the displacement. Blue and red meshes represent the lesion morphology at MRI time points 2 and 1, respectively. Note the asymmetric displacement inward from time point 1 (A1, purple mesh) as indicated by mixed directional vectors along with greater differences in the magnitude of change, leading to the resulting 3D shape at time point 2 for the MS lesion (A1, grey, A2). Compare to the SVD lesion with more uniform directional vectors outward from the original lesion acquired at time point 1 (**B**). B1. Green mesh demonstrating evolution in lesion size and relative preservation of the original shape at MRI time point 2. B2. Displacement vectors from the SVD lesion. Note the more uniform degree of change in magnitude and direction as compared to the MS lesion.

Figure 1 demonstrates both the magnitude and direction of displacement with resultant transitional shapes from the two MRI time points between disease states.

**Figure 2:**
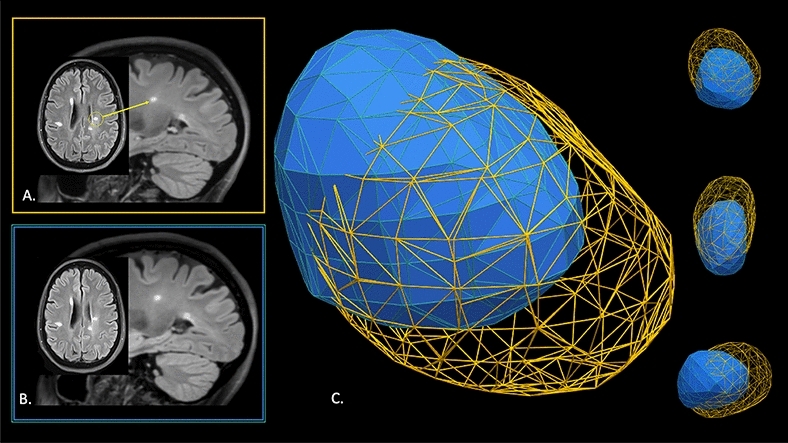
Two-dimensional (2D) MRI axial FLAIR and 3-dimensional (3D) sagittal FLAIR images highlighting a single MS lesion (circled in yellow) from a 49-year-old white woman with relapsing remitting disease from (**A**) time point 1 and (**B**) time point 2 (1-year follow-up). (**C**) Visual model in 3D demonstrating displacement and deformation of the lesion at time point 2 (blue) compared to the original position at time point 1 (yellow mesh). Note both the reduction in size and positioning of the lesion between time points that is not apparent on review of the longitudinal 2D MRI data (**A**,**B**).

The estimated median displacement for the SVD and MS cohorts for an average age patient with an average duration between scans and average surface area at baseline was 0.30 mm (95% CrI = (0.23, 0.40), Bayesian *p* value < 0.0001) and 0.40 mm (95% CrI = (0.34, 0.47), Bayesian *p* value < 0.001), respectively. Both of these findings revealed transitions in lesion displacement greater than 0 between MRI time points. A significant difference in the average log of the median displacement was not observed between groups (95% CrI = (− 0.06, 0.62), Bayesian *p* value = 0.10). However, as the difference between the two MRI time points increases by one standard deviation, the log of the median displacement increases by 0.21 mm (95% CrI = (0.05, 0.36), Bayesian *p* value = 0.007), suggesting greater shifts in the location of a lesion at the second MRI time point by 23.4% (95% CrI = 5.59%, 43.5%), as compared to the original position (Fig. 2).

### Deformation (evolution of shape between MRI time points)

**Figure 3:**
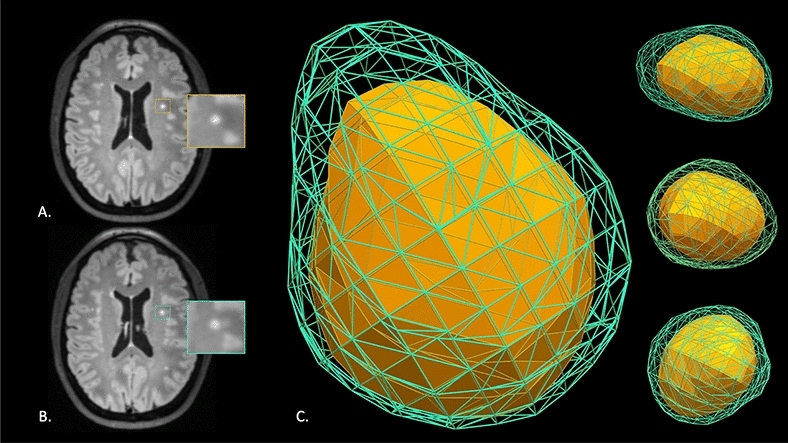
Two-dimensional (2D) MRI axial FLAIR brain images highlighting a single SVD lesion from a 42-year-old white woman from (**A**) time point 1 and (**B**) time point 2 (1-year follow-up). Visual simulation model in 3D demonstrating displacement and deformation of the lesion at time point 2 (fluorescent green) compared to the original position at time point 1 (solid yellow). (**C**) Note the symmetric and more uniform growth between time points in the simulation model that is not apparent when comparing high-resolution MRI data at 3-Tesla (**A**,**B**).

Based on our analysis of $R$ at time point 1, ${R}_{ij1}$, the data suggest that the posterior mean of (${R}_{ij1}-1)\times 100$ for an average age SVD and MS patient with an average surface area at baseline was 2.55 (95% CrI = 2.24, 2.90) and 3.17 (95% CrI = 2.89, 3.48), respectively. Therefore, a 24.6% greater deformation for the MS cohort relative to the SVD cohort was observed (95% CrI = (5.01%, 47.4%), Bayesian *p* value = 0.01). These results indicate a more spherical shape in the SVD cohort at MRI time point 1 relative to the MS cohort. Furthermore, for a lesion of average age with and average surface area at baseline, as the interval time between MRI studies increased by one year, the value of ((${R}_{ij2}-1)\times 100)$ did not significantly change relative to (${(R}_{ij1}-1)\times 100)$, indicating stability of the shape descriptor over time (95% CrI = (− 0.01, 0.22), Bayesian *p* value = 0.08). However, a significant decrease of 0.36 (95% CrI = (− 0.48, − 0.24), Bayesian *p* value < 0.0001) in spherical shape was observed for MS lesions, indicating instability of the shape descriptor over time. That is, the value of ((${R}_{ij2}-1)\times 100)$ decreased by 14.8% more than that of a SVD patient (95% CrI = (− 20.5%, − 8.87%), Bayesian *p* value < 0.0001). Therefore, lesions from SVD maintained a more spherical shape from the two MRI time points (Fig. 3).

A follow-up analysis was performed to determine if a threshold could be defined, in connection with ${R}_{ijt}$, to accurately classify MS versus SVD lesions. For the training model, a 92.7% in-sample accuracy and 91.2% out-of-sample accuracy was obtained if greater than 62.2% of the lesions within a given MRI study had a ($({R}_{ij1}-1) \times 100)$ value of > 2.49. These findings were consistent between both MRI time points 1 and 2 from MS and SVD patients in the validation studies with an in-sample and out-of-sample accuracy of 91.2% and 91.4%, respectively, at time point 1 and 91.2% and 94.3%, respectively, at time point 2.

## Discussion

MRI continues to remain a key tenet in diagnosis of MS, capable of fulfilling both dissemination in space and time criteria, and is invaluable in the surveillance of disease as MRI relapses outnumber clinical relapses^13^. The accurate classification of white matter anomalies observed on MRI leading to a diagnosis or response to treatment impression is paramount to ensure that patients receive the proper recommendations for management^14^. This issue is also of great importance for those MS patients in higher age categories as the origin of newly identified MRI lesions may be the result of SVD rather than new autoimmune inflammatory events^15^. Within the two MRI time points studied, we identified that MS and SVD lesions evolve significantly in three domains: volume, displacement or lesion position changes from origin, and shape consistency.

The method of studying how lesions evolve through defining morphological and spatial patterns of change provides an alternative approach towards determining the etiology of brain white matter anomalies. We identified a significant reduction in volume and a reduction in surface area trending towards significance in MS lesions. Lesions resulting from SVD were found to be less dynamic with no significant changes observed in these measures. The reduction in volume for MS lesions was consistent with previous observations of sustained “radiological contraction” in all lesions at an average rate of 4.5% annually over a mean study period of 16 years^16^. Our finding of a volume contraction was independent of the time interval between MRI studies, suggesting the promise for the early recognition of lesion changes between longitudinal MRI studies that may better indicate insights into cause. The observation of more modest surface area differences between MRI time points may be related to the more dynamic shapes associated with MS lesions, lesion age, or a combination of these factors^17,18^.

Beyond size and surface area metrics, we identified a significantly higher degree of displacement or distance deviation in lesion position from the origin in MS lesions when directly compared to SVD lesion transitions using a method employing 3D visual model data. The more dynamic transitions observed here may relate to physiological differences between the two lesion types with MS lesions being associated with greater imbalances in energy demand and supply with impaired mitochondrial energy production impacting ion homeostasis^19^, metabolic derangements with increased venous blood oxygenation compared to surrounding tissue^18^, inflammatory expansion^16^, and active remodeling or degenerative responses following injury^20^.

Our initial work revealed that MS lesions had a greater tendency to be asymmetric with complex surface features as compared to those resulting from SVD^17^. Given these data, we hypothesized that different patterns in lesion deformation or shape transformations exist between these two groups. By comparing the change between MRI time points to a reference shape, MS lesions demonstrated 34% greater deformation from a sphere relative to SVD lesions and our classification algorithm demonstrated robust accuracy rates in differentiating between these two lesion types when applied. The more spherical deformations observed with SVD, along with the lack of changes in lesion positioning, may point to the underlying pathophysiology of lesion development as hyaline degeneration of the subcortical arteries and arterioles along with resulting micro-ischemia, gliosis, and tissue degeneration dominate^21^. Alterations in endothelial shear stress, decreased vessel wall compliance, impaired vasodilation, changes in vessel thickness, perivascular enlargement, and amyloid ß peptide within vessels have also been implicated^2, 22–24^. These mechanisms of injury differ substantially with chronic autoimmune demyelinating events where persistent inflammation related to aberrant microglia or macrophage behavior may occur along with endogenous remyelination and secondary degenerative changes^25,26^. Collectively, these findings appear to be consistent with the lack of a shared mechanism for myelin injury that may result from a vascular or immune mediated process and may explain our observed difference in shape evolution^27^.

**Figure 4:**
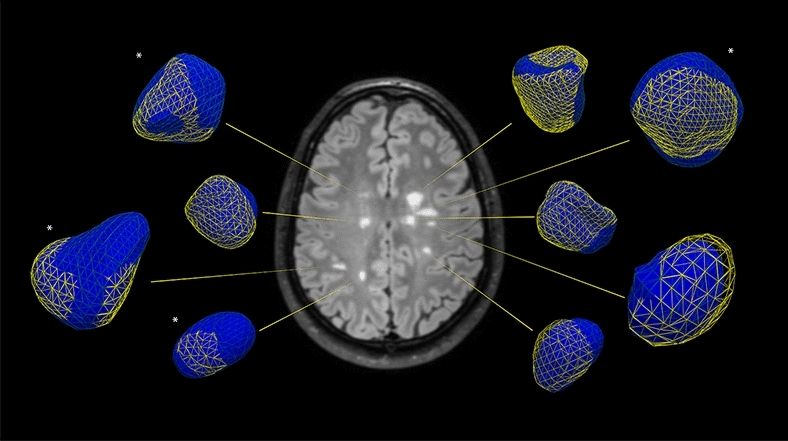
Two-dimensional (2D) MRI axial FLAIR image from a patient with relapsing remitting MS with corresponding 3-dimensional (3D) simulated lesions created from two MRI time points acquired approximately 1-year apart. The yellow-mesh represents the lesion in 3D at MRI time point 1 and solid blue, representing the evolution of the lesion at MRI time point 2. Note the heterogeneity of the 3D transitions with all lesions demonstrating displacement and a few revealing reductions or increases (‘smoldering’; *) in lesion volume.

Alternative approaches involving the evaluation of structural characteristics on MRI are needed to improve the specificity of lesion origin. The study of the evolution of 3D lesion shape transitions may effectively add to our understanding of lesion structure and MRI techniques to monitor disease by allowing for a determination of qualitative changes that are currently not discernable to the human eye when comparing MRI time points along with providing quantitative measures of variation. The approach may also reveal greater insights into disease activity by recognizing lesions prone to chronic active inflammation or ‘smoldering’ that are associated with clinical disability (Fig. 4) and have utility in situations where the diagnosis of inflammatory demyelination may be equivocal or suggestive of radiologically isolated syndrome (RIS)^9, 12,28–31^.

The findings here should be evaluated in the context of important limitations. A larger sample size involving the study of more patients and lesions would be ideal with more uniform timing between scans and longer follow-up times, however our age and time-adjusted 3D metrics still exhibited robust differences between groups. Although our outcomes were independent of disease modifying therapy exposure, the impact of distinct disease modifying therapies when studied in larger numbers may prove to be meaningful as higher efficacy agents may lead to more dynamic changes in lesion morphology. In addition, in its current state, the 3D technology utilized here is limited to focal lesions. Less is known regarding longitudinal morphological changes resulting from T2-hyperintensities that coalesce over time. Furthermore, the impact of whole brain or regional changes in volume (i.e. white matter volume) in relation to structure is unknown, although heterogeneity in lesion behavior is apparent even within a single hemisphere and more refined local measures of white matter change may better inform on the impact on 3D lesion structure rather than global measures (Fig. 4). Lastly, despite the strength of the statistical models used for our analysis, the identification of lesion-specific covariates (i.e. true lesion age, impact of paramagnetic rims^12^, etc.) for future studies will be needed to increase the accuracy of the predictive models utilized due to the large degree of within-patient heterogeneity.

The approach of studying the physical 3D characteristics of supratentorial lesions appears to offer a higher fidelity metric to conventional imaging data when evaluating for interval change between MRI time points. To realize the importance of the evolution of size, surface area, lesion shape and displacement and the implications for our patients, future studies are needed to define the interdependence between these structural changes and longer-term clinical outcomes. The value of quantifying lesion transitions in morphology and positioning may address fundamental questions regarding lesion behavior in vivo, and we speculate that such findings may eventually result in a remarkable change in our approach towards the diagnosis and management of MS.

## Methods

### Research participants

The study group was ascertained from patients evaluated in the Clinical Center for Multiple Sclerosis at the University of Texas Southwestern (UTSW) Medical Center. Inclusion criteria were comprised of (1) male or female patients ≥ 18 years of age with either (2) an established diagnosis of relapsing–remitting MS following a comprehensive medical evaluation by fellowship trained MS specialists without history of migraine, use of recreational substances, or significant vascular risk factors, (3) absence of an acute neurological exacerbation related to CNS demyelination 30 days prior to the first MRI study and within the time interval between MRI time points, (4) lack of exposure to oral or intravenous glucocorticosteroid treatment 30 days prior to the first MRI study and no exposure during the time interval between scans, (5) no change in treatment assignment within 90 days prior to the first MRI and in the interval between MRI time points, or vi) confirmed non-MS patients with a history of migraine headaches or small vessel disease risk factors with focal brain MRI white matter abnormalities present within the bilateral supratentorial white matter. Exclusion criteria included (1) female patients who were pregnant or lactating, (2) severe claustrophobia, and (3) reduced quality of MRI data limiting the 3D image processing.

Recruited patients were placed into two groups: (1) patients with a confirmed diagnosis of MS based on established criteria^1^, results from supporting para-clinical studies (i.e. cerebrospinal fluid profiles, electrophysiological data, serological results) and the exclusion of other disease states, and (2) patients with a history of brain anomalies atypical for inflammatory demyelination based on the observed radiological phenotype and formal imaging interpretations by board certified neuroradiologists and clinical impressions by specialists in MS. Conclusions were further supported by negative laboratory, genetic, or other para-clinical studies to identify a clear underlying etiology.

The protocol was approved by the Institutional Review Board at UTSW Medical Center, and informed consent was obtained from all study participants. In addition, this study was performed in accordance with the Code of Ethics of the World Medical Association (Declaration of Helsinki) and all relevant institutional guidelines and regulations.

### Image acquisition

All imaging studies were performed on a 3T MRI scanner (Philips Medical Systems, Cleveland, OH) using a 32-channel phased array coil for reception and body coil for transmission. Each MRI study included scout localizers, 3D high-resolution inversion recovery spoiled gradient-echo T1-weighted isotropic (1.0 × 1.0 × 1.0 mm^3^, TE/TR/TI = 3.7/8.1/864 ms, flip angle 12 degrees, 256 × 220 × 170 mm^3^ FOV, number of excitations (NEX) = 1, 170 slices, duration: 4:11 min), 3D fluid-attenuated inversion recovery (FLAIR) (1.1 × 1.1 × 1.1 mm^3^, TE/TR/TI = 350/4800/1600 ms, flip angle 90 degrees, 250 × 250 × 180 mm^3^ FOV, NEX = 1, 163 slices, duration: 5:02 min) and 3D T2-weighted sequence acquired in sagittal plane (1.0 × 1.0 × 1.0 mm^3^, TE/TR/TI = 229/2500/1600 ms, flip angle 90 degrees, 250 × 250 × 180 mm^3^ FOV, NEX = 1164 slices, duration: 4:33 min).

### Lesion segmentation

Analyses were implemented without knowledge of clinical history, current or past treatments, or disease duration. MRI studies from the two time points were initially registered based on anatomical positioning and intensity using an in-house developed software package previously utilized in prior studies, Med-IP^18,32^.

MRI studies were aligned using a modified version of Insight Tool Kit (ITK) multi-resolution rigid registration with Mattes Mutual Information Metric. Intensity alignment was performed locally around each lesion across the two time points through histogram matching. The histogram matching technique employed utilized a set of linear transforms computed from ordered correspondence on a set of match points from the quantiles of each histogram of the local region around each lesion, ensuring local consistency across MRI time points for each lesion^33^. After positional and local intensity alignment, segmentations on selected focal supratentorial brain lesions measuring ≥ 3 mm^2^ that were verified from simultaneously viewed 3D high-resolution FLAIR and T2-weighted sequences were performed by implementing geodesic active contouring methodology with manual editing^34^.

Quality assurance assessments were also performed to ensure the accuracy of lesion segmentations. Quantitative data analyses including volume, surface area, displacement, and deformation calculations were then performed using Med-IP on individual and registered image files from both MRI time points.

### 3-dimensional conformation metrics

#### Volume and surface area

For each lesion segmented at each MRI time point, lesion volume and surface area measurements were calculated. Changes in lesion volume and surface area over time were also determined.

### Displacement (change in lesion positioning)

Three-dimensional image analysis was used to compute a metric defined as displacement which quantified changes in the space occupied by a given lesion between the two time points. For each lesion at each time point, a multitude of points were designated on the lesion surface at the first time point and their displacement at the second time point computed. As the number of points designated on the lesion surface is dependent upon lesion size, a range from 18 points to 2620 points in our dataset was observed. Due to the differing number of points between lesions and the desire to simplify the analysis from a multivariate problem with lesion-specific dimensionality to a univariate problem, the median displacement was computed for each lesion. Furthermore, the median displacement was log-transformed to obtain a suitable model fit.

### Deformation (evolution of shape between MRI time points)

Based on previously observed 3D renderings of lesions from MS patients, it became apparent that SVD lesions exhibited a more spherical shape relative to MS lesions. Therefore, we developed a metric which informs on the resemblance of a lesion to a sphere based on the previously computed volume and surface area. For a sphere with radius $r$, ${\text{volume}}=4\pi {r}^{3}/3$ and $\text{surface area}=4\pi {r}^{2}$. Moreover, the radius can be computed as a function of volume,

1 $${r}^{Vol}= \sqrt[3]{0.75\cdot {\text{Volume}}/\pi ,}$$

or as a function of the surface area,

2 $${r}^{SA}=\sqrt{0.25\cdot \text{Surface Area}/\pi .}$$

If a given lesion is truly a sphere, we would expect ${r}^{Vol}={r}^{SA}$, or ${r}^{SA}/{r}^{Vol} = 1$. Furthermore, given that the ratio of surface area to volume is minimized for a sphere and becomes greater for more complex shapes, if we compute the ${r}^{Vol}$ and ${r}^{SA}$ based on Eqs. (1) and (2) for any 3D shape, then $R={r}^{SA}/{r}^{Vol} \ge 1$, with values of $R$ > 1 indicating greater deviations from a spherical shape. We have defined this metric as deformation relative to a sphere, or simply deformation. Let ${R}_{ijt}$ denote the value of $R$ for the $i$-th patient with $j$-th lesion at time point $t$, $t=1, 2$.

A classification algorithm was developed in which a threshold for $R$ and threshold for the proportion of lesions from a given patient greater than the threshold for $R$ were estimated based on area under the receiver operating characteristic curve. Leave-one-out cross-validation was performed to assess the predictive ability of the proposed classification algorithm. Both time points for each subject were used in the classification algorithm. The classification algorithm was trained using $2\times n-1$ patients (training sample) that predicted not only the sample used to train the algorithm, but also the “testing sample” represented by 1 patient left out at each iteration. The average accuracy of the training sample predictions (i.e., in-sample accuracy) and testing sample prediction (i.e., out-of-sample accuracy) were also determined.

### Statistical analysis—Lesion characteristic data

Bayesian linear (or generalized linear) mixed effects models were used to model the differences in the 3D morphology metrics between the MS and SVD cohort, controlling for age and time between MRIs. Additionally, in the analysis of volume change over time, a covariate was also included to account for volume at baseline. Similarly, in the analysis of surface area change, displacement, and deformation, a covariate was also included to account for surface area at baseline. Surface area was chosen as a covariate in the models for displacement and deformation, instead of volume, given a greater correlation between surface area and displacement or deformation. Mixed effects models were implemented to capture intra-subject and, if applicable, intra-lesion correlation. Because of the limited amount of prior data involving similar work, weakly informative prior distributions were used for all models.

### Software and model fitting

Bayesian analysis was performed using RStan^35^ in R^36^. In order to ensure convergence within the model, 3 chains using 15,000 iterations with a 5000 iteration burn-in were run. Convergence was examined using the trace plots of parameters in the model. Once convergence to a stationary distribution was verified, the model was run with a single chain containing 15,000 iterations with a 5000 iteration burn-in. Distributional assumptions for the response were assessed by plotting the sorted posterior mean of the residuals against the mean of the ordered posterior predictive residuals. A plot of the posterior mean of the residuals versus the posterior mean of the fitted values was generated to examine the homogeneity of variance assumptions for models assuming normal or Student’s *t*-distributed errors. Lastly, a plot of the prediction error generated based on the posterior predictive distribution versus the observed values of the response was generated.

A two-sided Bayesian *p* value was computed and a *p* value < 0.05 was considered significant for all statistical tests. The posterior mean of the parameters-of-interests were presented as the estimate of the parameter values, along with the credible interval.
